# Solitary Superficial Angiomyxoma in an Uncommon Location: A Case Report and Literature Review

**DOI:** 10.7759/cureus.67521

**Published:** 2024-08-22

**Authors:** Alexandra Denisa Oprea, Irina Margaritescu, Cristina Beiu, Diana Radaschin, Marius Popescu, Liliana Gabriela Popa

**Affiliations:** 1 Dermatology, Elias Emergency University Hospital, Bucharest, ROU; 2 Dermatopathology, OncoTeam Diagnostic, Bucharest, ROU; 3 Dermatology, Carol Davila University of Medicine and Pharmacy, Elias Emergency University Hospital, Bucharest, ROU; 4 Dermatology, Saint Parascheva Infectious Disease Clinical Hospital, Faculty of Medicine and Pharmacy, “Dunarea de Jos” University of Galati, Galati, ROU; 5 Physical Medicine and Rehabilitation, Carol Davila University of Medicine and Pharmacy, Elias Emergency University Hospital, Bucharest, ROU

**Keywords:** mohs surgery, spindle-shaped cells, myxoma, carney complex, superficial angiomyxoma

## Abstract

Superficial angiomyxoma (SAM) is a rare, benign, and slow-growing soft tissue tumor with a tendency for frequent local recurrence. Most SAMs are solitary and sporadic. However, multiple SAMs, particularly on the external ear or eyelids, may be the initial or only sign of the Carney complex, an autosomal dominant syndrome that impacts various organs, including the heart, breasts, and skin, and is linked to endocrine hyperactivity. To ensure an accurate diagnosis, a comprehensive patient interview, physical examination, and laboratory tests, including endocrine-specific markers and imaging studies, are essential. Due to the high risk of recurrence, especially in large, encapsulated lesions, complete surgical excision is the preferred treatment approach. We present a case of a 24-year-old female with SAM on the shoulder, review the relevant literature, and discuss the pathogenesis and appropriate management of such cases.

## Introduction

Superficial angiomyxoma (SAM), first described in 1985, is a relatively under-recognized cutaneous tumor. In 1988, Allen et al. classified angiomyxomas into two subtypes: aggressive and superficial [1]. Aggressive angiomyxoma, a rare myxoid neoplasm, predominantly affects the genital, perineal, and pelvic regions in young women, suggesting estrogen’s role in its growth [2]. In contrast, SAM can occur at any age, with a slight male predominance, and is most common in the third to fourth decade of life. Pediatric cases, particularly in the genital area, are rare [2]. Angiomyofibroblastoma, added later to the classification, is now considered a third subtype of angiomyxoma [3].

SAM typically appears as a slow-growing, asymptomatic solitary nodule or polypoid lesion less than 5 cm in diameter, often with a multilobular or multinodular form. It most frequently arises on the trunk, head and neck, and lower extremities and rarely affects the breasts or eyelids.

The presence of multiple SAMs should prompt consideration of the Carney complex, a rare syndrome featuring cutaneous myxomas, mammary myxomas, cardiac myxomas, lentigines, endocrine hyperactivity, and psammomatous melanotic schwannoma. SAM may be the earliest or sole manifestation of this syndrome.

Immunohistochemical studies indicate that loss of PRKAR1A protein expression is involved in SAM development [4]. This loss may result from genetic defects, epigenetic abnormalities (such as DNA hypermethylation), or protein-related deviations (like posttranscriptional modifications). Thus, PRKAR1A immunostaining is a crucial diagnostic tool, particularly for distinguishing SAM from other myxoid lesions in small biopsy specimens [4].

Given the high rate of local recurrence (30-40% after incomplete resection) [5], complete surgical excision is the treatment of choice. Mohs micrographic surgery may be considered for difficult-to-treat areas where preserving tissue for cosmetic reasons is essential.

## Case presentation

**Figure 1:**
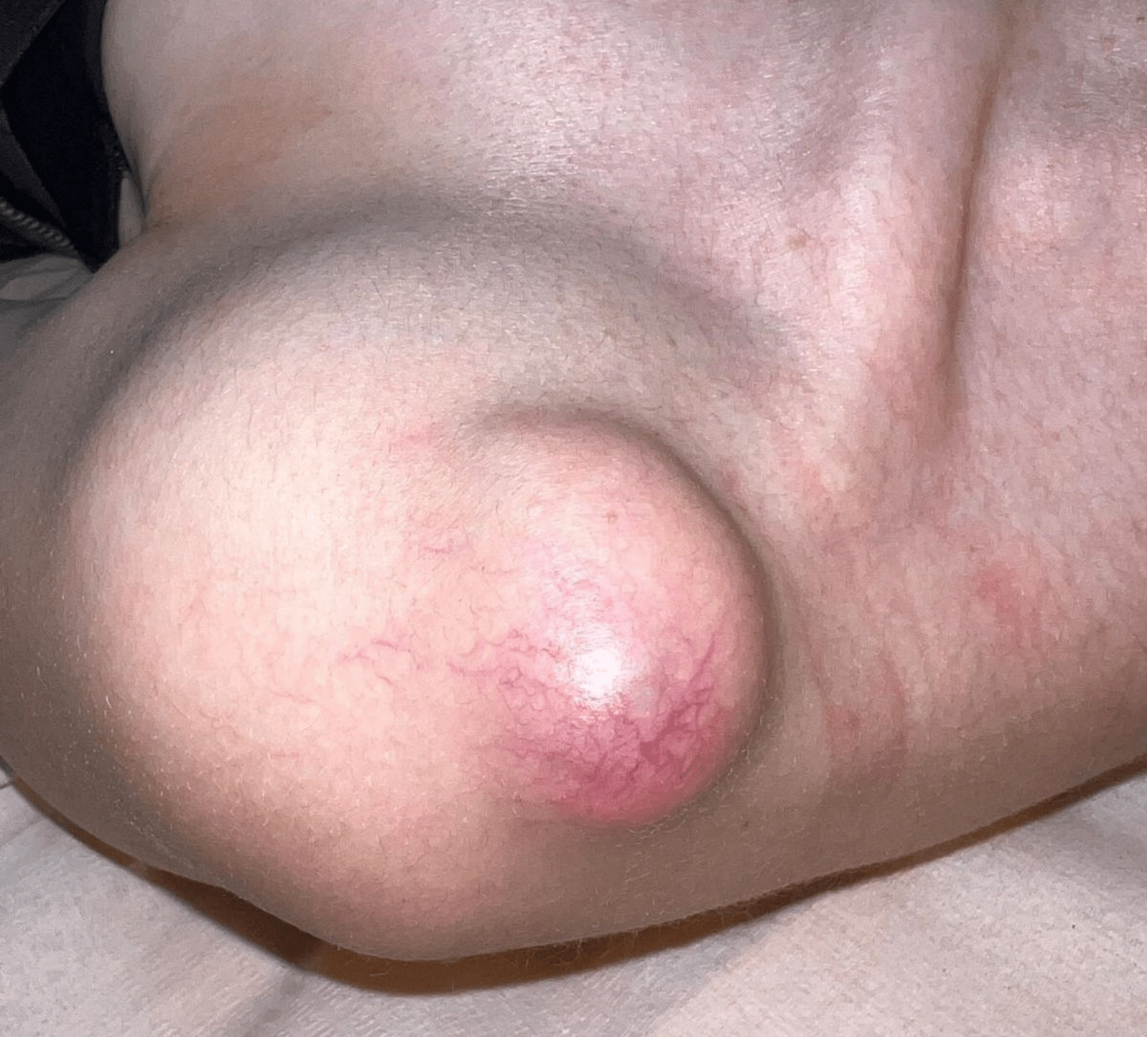
Well-defined, skin-colored nodule with fine superficial vessels on the surface, measuring 7 cm in diameter. The nodule is located on the left shoulder and is surrounded by normal skin.

A 24-year-old female, with no significant personal or familial medical history, presented to our clinic with a slowly enlarging nodule on her left shoulder that had been present for one year. The skin examination revealed a well-defined, skin-colored nodule with fine superficial vessels on the surface, measuring 7 cm in diameter, surrounded by normal skin (Figure 1). The nodule was tender upon palpation.

**Figure 2:**
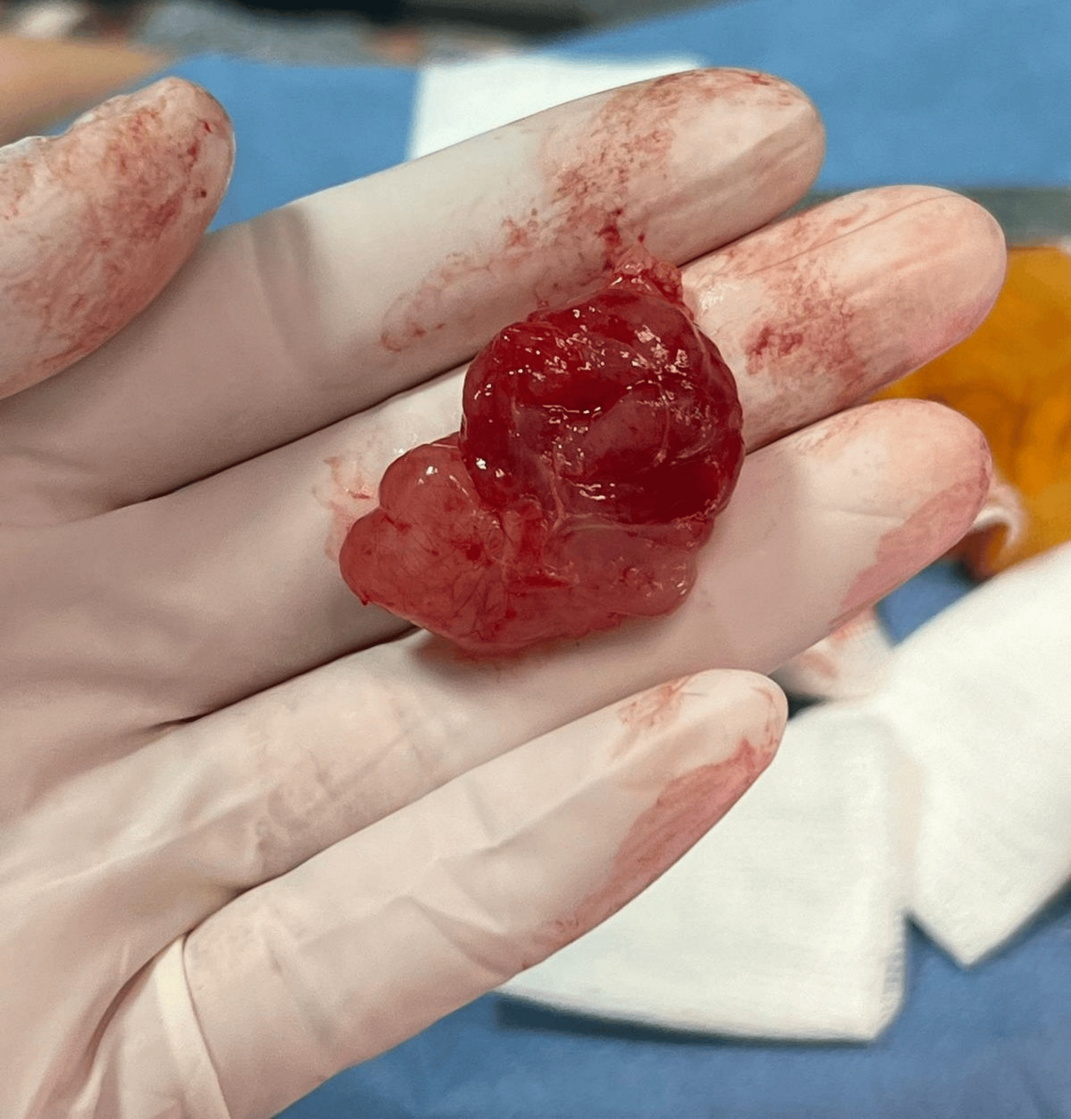
Macroscopic appearance of a partially encapsulated, highly vascular cutaneous tumor. The tumor displays a smooth surface, bosselated contours, and a pink color, measuring 5 × 4 cm.

**Figure 3:**
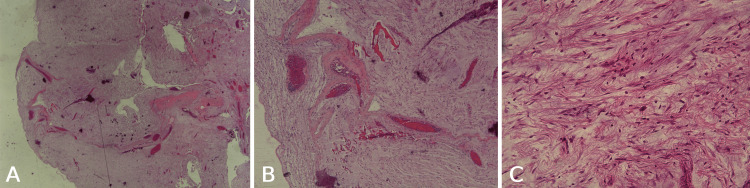
H&E stain. The H&E stain reveals a well-defined, encapsulated tumor mass with monomorphous spindle-shaped cells, small nuclei, reduced cytoplasm, and thin-walled blood vessels embedded in abundant myxoid stroma. The tumor shows no pleomorphism or nuclear atypia (H&E, A: 12.5×, B: 25×, and C: 200×).

An excisional biopsy was performed (Figure 2), and the histopathologic examination revealed a well-defined, encapsulated tumor mass. The tumor exhibited monomorphous spindle-shaped cells with small nuclei, prominent nucleoli, reduced cytoplasm, and thin-walled blood vessels embedded in a myxoid stroma. No pleomorphism, nuclear atypia, or necrosis was observed (Figure 3). Tumor cells stained intensely positive for CD34 and were only focally positive for actin, but negative for SOX10, calponin, and MUC4. The Ki-67 proliferation index was 10-12%. To prevent recurrence, the patient underwent a wide surgical excision.

Due to the particularities of the case - a large tumor, more than 5 cm in diameter, in contact with bone affecting a young female patient - she was referred to an endocrinologist, and additional laboratory analyses were conducted to rule out endocrine hyperactivity. Levels of intact parathormone, vitamin D, prolactin, growth hormone, insulin-like growth factor 1, and thyroid-stimulating hormone were within normal limits. Additionally, cardiac and thyroid ultrasounds did not reveal any pathological findings. Consequently, the Carney complex was ruled out.

## Discussion

Systematic literature review

**Figure 4:**
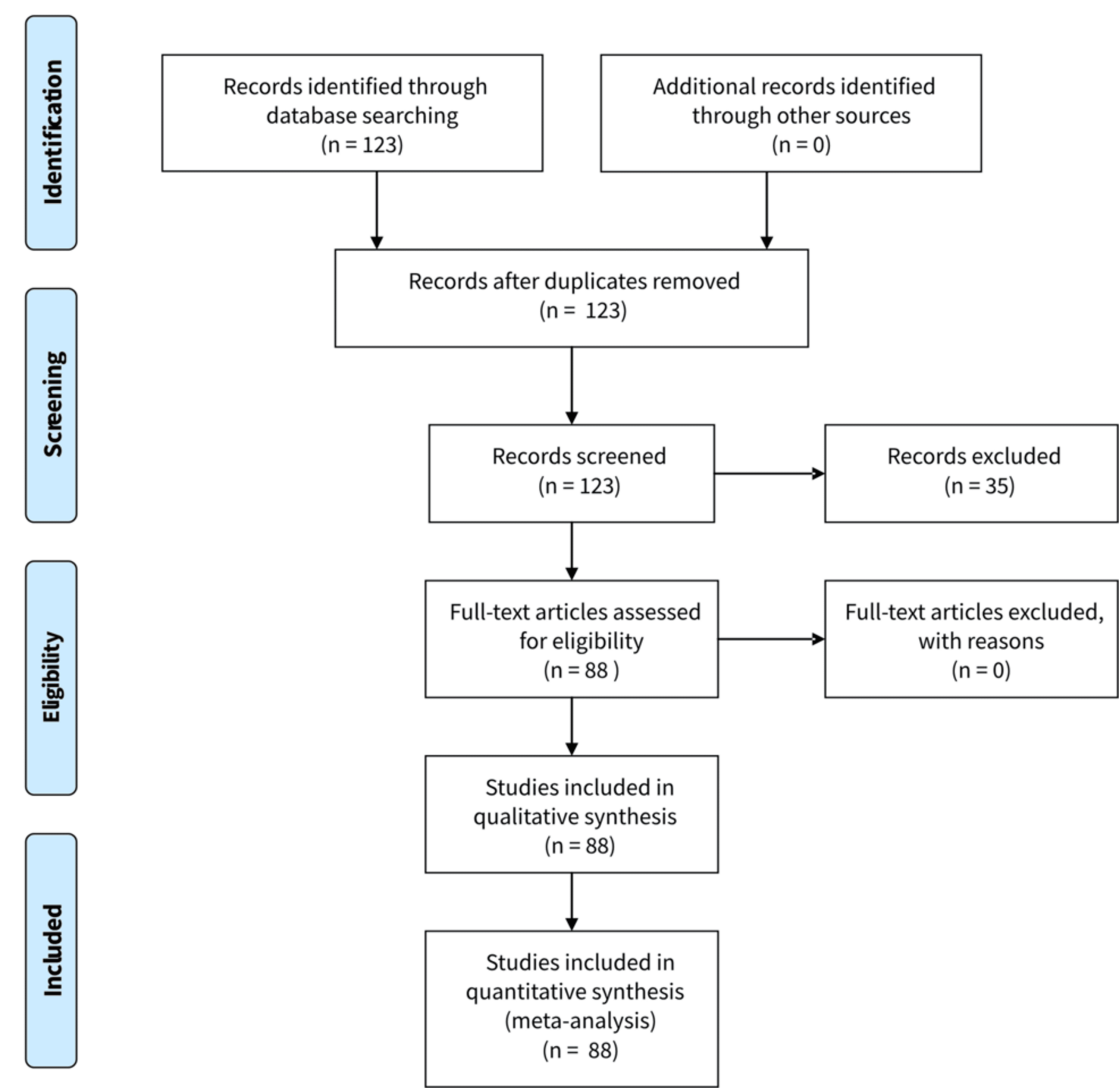
PRISMA flow diagram. Of the 123 records identified through database searching, 35 were review articles and were excluded. The remaining 88 records, detailing SAM cases, were included in the qualitative and quantitative synthesis. PRISMA, Preferred Reporting Items for Systematic reviews and Meta-Analyses; SAM, superficial angiomyxoma

To better understand the epidemiology and progression of SAM, we conducted a review of the medical literature. We searched for the term “solitary angiomyxoma” using PubMed and Google Scholar, identifying 88 relevant publications (Table 1) [1-88]. We analyzed data from these studies, focusing on lesion location, age at diagnosis, patient gender, and recurrence rates during follow-up. The review followed Preferred Reporting Items for Systematic reviews and Meta-Analyses (PRISMA) guidelines (Figure 4) [89]. Out of 123 records identified, 35 were review articles and were excluded. The remaining 88 records, detailing SAM cases, were included in both the qualitative and quantitative analyses.

**Table 1:** SAM cases reported in the medical literature. SAM, superficial angiomyxoma; UA, unavailable information

No.	Study	Number of cases	Location	Age of patients at diagnosis	Gender	Course
1	Sharma et al. [6]	54	19 on the trunk, especially on the back; 18 on the lower limbs; 10 on the head and neck region; 7 on the arm	7 to 91 years (mean, 55 years)	31 F, 23 M	1 tumor recurred after 3 years
2	Baranov et al. [7]	40	All tumors are located on the breasts, 9 of them on the nipple-areola complex	14 to 72 years (mean, 40 years)	22 F, 18 M	1 tumor recurred after incomplete excision
3	Calonje et al. [8]	39	17 on the trunk, 14 on the head and neck, and 7 on the lower limbs	Birth to 82 years (median, 45.5 years)	25 M, 14	8 tumors recurred, 4 of them twice
4	Allen et al. [1]	30 tumors in 28 patients	11 on the trunk, 10 on the lower extremity, 5 on the head or neck, and 4 on the arm	4 to 78 years (mean, 39 years)	16 M, 12 F	8 tumors recurred once 1 patient developed a new SAM
5	Neumann et al. [4]	28	UA	5 to 87 years (mean 49.7 years)	UA	UA
6	Fetsch et al. [9]	17	M: all lesions were located on the scrotum; F: 6 on the labium, 4 on the vulva, 2 on the groin, and 1 on the mons pubis	M: 18 to 55 years (mean: 39 years), F: 15 to 33 years (mean: 21 years)	4 M, 13 F	3 tumors recurred after 8 months, 7 years, and 20 years
7	Perret et al. [10]	4	Face lower limb subungual subungual	55, 61, 18, 53	F, F, M, M	UA
8	Nakayama et al. [11]	1	Inguinal region	64	M	UA
9	Velanovich [12]	1	Inguinal region	2	M	UA
10	Teixeira-De-Magalhães and Pardal-De-Oliveira [13]	1	Larynx	UA	UA	Recurred
11	Izquierdo et al. [14]	1	Areola	49	F	UA
12	Ustun et al. [15]	1	Vulva	42	F	UA
13	Bedlow et al. [16]	1	Scalp	Congenital	F	UA
14	Chen et al. [17]	1	Buccal mucosa	19	M	UA
15	Vella et al. [3]	1	Epididymis	50	M	UA
16	Gardner [18]	1	Floor of the mouth	69	F	UA
17	Yamamoto et al. [19]	1	Lower limb	19	M	UA
18	Rodríguez-Vázquez et al. [20]	1	Preauricular region	34	F	UA
19	Yuen et al [21]	1	Eyelid	47	M	UA
20	Okada et al. [22]	1	Vulva	3	F	UA
21	Misago et al. [23]	1	Digit	59	F	UA
22	Wu and Lin [24]	1	Neck	26	M	UA
23	Kim et al. [25]	1	Vulva	26	F	UA
24	Satter [26]	1	Midback	35	M	UA
25	Pérez Tato et al. [27]	1	Trunk	51	M	UA
26	Meer and Beavon [28]	1	Buccal mucosa	37	F	UA
27	Khadilkar et al. [29]	1	External ear	20	F	UA
28	Nakamura and Tokura [30]	1	Scrotum	4	M	UA
29	Rosado Rodríguez et al. [31]	1	Parotid region	61	M	UA
30	Zhu et al. [32]	1	Vulva	27	F	UA
31	Toth et al. [33]	1	Retropharyngeal	28	M	UA
32	Qian et al. [34]	1	Toe	60	M	UA
33	Ali et al. [35]	1	Eyelid	77	M	Recurred after 8 years
34	Falidas et al. [36]	1	Subungual	45	M	UA
35	Basak et al. [37]	1	Vulva	39	F	Recurred 18 months after incomplete resection
36	Nishio et al. [38]	1	First web space of the hand	39	M	UA
37	Sibley et al. [39]	1	Scalp	9	M	UA
38	Zhu et al. [40]	1	Vulva	27	F	UA
39	Ravindra et al. [41]	1	Upper posterior alveolar mucosa	30	M	UA
40	Diniz et al. [42]	1	Gluteal region	12	F	UA
41	Lee et al. [5]	1	Scrotum	56	M	UA
42	Wang et al. [43]	1	Scrotum	25	M	UA
43	Kahn et al. [44]	1	Nasal dorsum	28	M	UA
44	Kura et al. [45]	1	Digit	UA	UA	UA
45	Green et al. [46]	1	UA	33	M	UA
46	Bhat et al. [47]	1	Digit (thumb)	70	M	UA
47	Zhang and Xu [48]	1	UA	UA	UA	UA
48	Lemtibbet et al. [49]	1	Plantar region	60	F	UA
49	Ozdemir et al. [50]	1	Vulva	26	F	UA
50	Victoria Martínez et al. [51]	1	Nipple	28	F	UA
51	Shukla et al. [52]	1	Parotid region	congenital	F	UA
52	Lee et al. [53]	1	Vulva	60	F	UA
53	Abarzúa-Araya et al. [54]	1	Lower back	72	M	UA
54	Aberdein et al. [55]	2	Scalp, lower back	63, 47	M, M	UA
55	Anehosur et al. [56]	1	Buccal vestibule	32	M	UA
56	Singhota et al. [57]	2	Lower lip palate	47, 58	F, M	UA
57	Wang et al. [58]	2 tumors in 1 patient	Penis	18	M	1 tumor recurred 2 years after incomplete excision
58	Imen et al. [59]	1	Vagina	40	F	UA
59	Bajpai et al. [60]	1	Palate	29	M	UA
60	Hamzelou et al. [61]	1	Plantar region	25	M	UA
61	O'Flynn O'Brien et al. [62]	1	Vulva	7	F	UA
62	Iwashita et al. [63]	1	Nipple	39	F	UA
63	Chen et al. [64]	1	Vulva	50	F	UA
64	Hafeez et al. [65]	1	Vulva	1.5	F	UA
65	Hwang et al. [66]	1	Posterior neck	6	M	UA
66	Amores-Martín et al. [67]	1	Lower limb	62	M	UA
67	Chen et al. [68]	1	Nasal vestibule	53	F	UA
68	Hosapatna Basavarajappa et al. [69]	1	Vulva	25	F	UA
69	Morimoto et al. [70]	1	Gingiva	15	F	UA
70	Fotiadou et al. [71]	1	Tongue	70	F	UA
71	Dubin et al. [72]	1	Breast	16	F	UA
72	Xu et al. [73]	1	Scrotum	N/A	M	UA
73	Chen et al. [74]	1	Eye socket	23	M	UA
74	Meng et al. [75]	1	Breast	25	F	UA
75	Yan et al. [76]	1	Perineum	42	M	UA
76	Chijiiwa et al. [77]	1	Wrist	71	M	UA
77	Ros Briones et al. [2]	1	Vulva	congenital	F	UA
78	Hirai et al. [78]	1	Soft palate	41	M	UA
79	Navea et al. [79]	1	Popliteal fossa	61	M	UA
80	Tonape and Sv [80]	1	Axilla	42	M	UA
81	Park et al. [81]	1	Nipple	12	F	UA
82	Singh et al. [82]	1	Ear pinna	24	F	UA
83	Navitski et al. [83]	2	Vulva	28, 42	F, F	UA
84	Mehrotra et al. [84]	2	Vulva	38, 17	F, F	UA
85	Yun et al. [85]	1	Eyelid	29	F	UA
86	Bembem et al. [86]	1	Lower limb	14	F	UA
87	Lee et al. [87]	1	Vulva	13	F	UA
88	Quatresooz et al. [88]	1	Submammary region	40	F	UA

Out of the 88 publications, seven presented case series with four to 54 patients [1,4,6-10], while the remaining were case reports. In total, 298 SAMs have been reported in 295 patients. Cases were reported across all ages, including three congenital SAMs in female patients located on the scalp, parotid region, and vulva. The oldest patient was 91 years old, with a mean age of 37.9 years. Of the 295 patients, 150 were female, resulting in a female-to-male ratio of 1.03.

**Figure 5:**
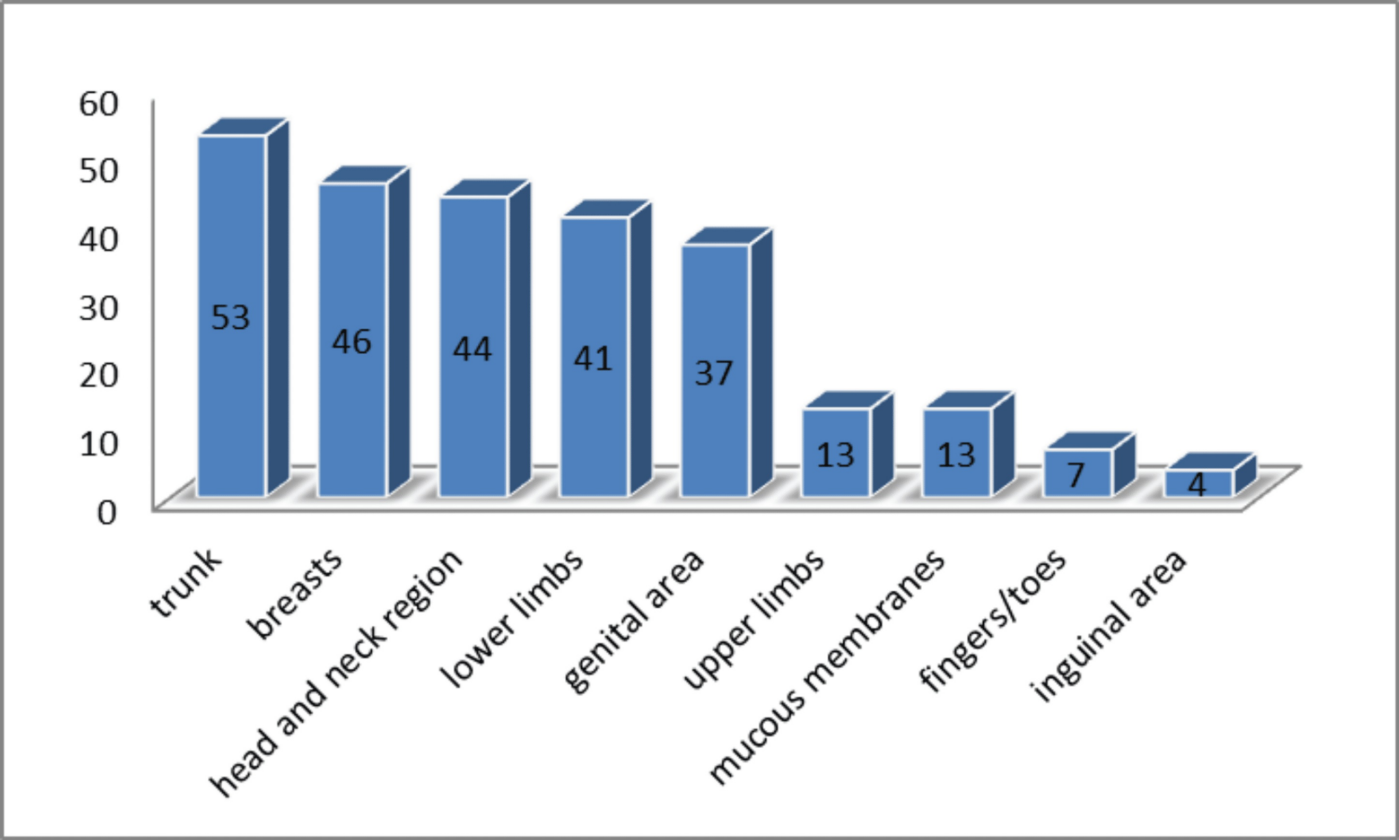
Distribution of SAM cases reported in the literature according to tumor location. SAM, superficial angiomyxoma

Tumor locations included 53 cases (17.7%) on the trunk, particularly the lower back; 46 cases (15.4%) on the breasts, with 13 (4.3%) involving the nipple-areola region; 44 cases (14.7%) in the head and neck region, including three on the eyelid, one in the eye socket, two on the external ear, three in the parotid region, and three on the scalp; 41 cases (13.7%) on the lower limbs, including two on the soles (0.6%); 37 cases (12.4%) in the genital area, with tumors in the vulva (27 cases), vagina (one case), scrotum (eight cases), penis (one case), and epididymis (one case). Less common sites included the upper limbs (13 cases or 4.3%), fingers/toes (seven cases or 2.3%, including three subungual SAMs), and the inguinal region (four cases or 1.3%). SAMs also developed in the oral mucosa (10 cases or 0.3%), retropharyngeal area (one case or 0.3%), larynx (one case or 0.3%), and nasal vestibule (one case or 0.3%) (Figure 5).

The majority of patients underwent complete surgical excision, although Mohs micrographic surgery, shave biopsy, and laser treatment were also used. Follow-up intervals varied from six months to two years. Recurrence occurred in 25 tumors, often after incomplete excision, with four tumors recurring twice. The time to recurrence ranged from eight months to 20 years.

SAM is a rare, slow-growing benign soft tissue tumor with a significant risk of local recurrence. Diagnosis relies on clinical presentation and histopathologic features, though additional diagnostic methods can be helpful. Dermoscopy may reveal the “red planet sign” - reddish translucent globules with fine superficial vessels [46]. Ultrasonography can assist in the initial diagnosis by showing an irregular, heterogeneously echogenic cystic and solid lesion with multiple thin echogenic septa and micro-lobulated margins [5,64]. CT scans show similar heterogeneity with variable enhancement, while MRI typically shows a homogeneous tumor with fluid-like signal intensity and variable heterogeneous enhancement on contrast-enhanced images [5].

SAM can mimic various benign and malignant tumors, necessitating careful clinical and histopathological differential diagnosis. Differential diagnoses include aggressive angiomyxoma, myxoid neurofibroma (SOX10 positive), soft tissue myxoma (characterized by prominent myxoid matrix), nodular fasciitis (with spindle stellate cells and positive calponin staining) [86,90], and low-grade myxofibrosarcoma (with abundant mucoid stroma, an arborizing capillary network, lipoblasts, and MUC4 positivity) [86,90]. Myxoid dermatofibrosarcoma, which shows spindle cells with glassy eosinophilic cytoplasm, vesicular nuclei, and focal CD34 positivity, should also be considered [86,90].

Despite its benign nature, SAM’s high recurrence rate post-excision and potential for rare muscle infiltration underscore the importance of Mohs micrographic surgery for small tumors to ensure clear margins. Larger, encapsulated lesions often require traditional excision. Long-term follow-up is essential, as recurrences may appear many years after the initial resection.

## Conclusions

Due to its rarity and subtle histological features, SAM necessitates thorough evaluation by experienced pathologists and careful correlation with clinical findings (age, gender, location, and symptoms) for an accurate diagnosis. Additional diagnostic tools, such as dermoscopy, ultrasonography, CT, and MRI, can aid in differential diagnosis. A complete surgical excision is crucial for achieving a favorable aesthetic and functional outcome, given the tumor’s significant potential for local recurrence, which can occur even decades after the initial diagnosis. We aim to raise awareness about this uncommon tumor and emphasize the importance of close monitoring for affected patients.

## References

[1] Allen PW, Dymock RB, MacCormac LB (1988). Superficial angiomyxomas with and without epithelial components. Report of 30 tumors in 28 patients. Am J Surg Pathol.

[2] Ros Briones R, Arredondo Montero J, Bronte Anaut M, Hernández-Martín S, Guarch Troyas R (2023). Pediatric vulvar superficial angiomyxoma: a case report with clinical, radiological, and anatomopathological characterization and a comprehensive review of the literature. Int J Surg Pathol.

[3] Vella R, Calleri D (2000). Superficial angiomyxoma of the epidiymis. Presentation of a new case and clinical considerations [Article in Italian]. Minerva Urol Nefrol.

[4] Neumann NM, LeBoit PE, Cohen JN (2022). Superficial angiomyxomas frequently demonstrate loss of protein kinase a regulatory subunit 1 alpha expression: immunohistochemical analysis of 29 cases and cutaneous myxoid neoplasms with histopathologic overlap. Am J Surg Pathol.

[5] Lee CU, Park SB, Lee JB, Park HJ, Kim MK, Chang IH (2015). Sonographic findings of prescrotal superficial angiomyxoma. Jpn J Radiol.

[6] Sharma A, Khaitan N, Ko JS (2022). A clinicopathologic analysis of 54 cases of cutaneous myxoma. Hum Pathol.

[7] Baranov E, Alston EL, Lester SC, Fletcher CD, Doyle LA (2023). Angiomyxoma of the breast: a clinicopathologic analysis of 40 cases. Am J Surg Pathol.

[8] Calonje E, Guerin D, McCormick D, Fletcher CD (1999). Superficial angiomyxoma: clinicopathologic analysis of a series of distinctive but poorly recognized cutaneous tumors with tendency for recurrence. Am J Surg Pathol.

[9] Fetsch JF, Laskin WB, Tavassoli FA (1997). Superficial angiomyxoma (cutaneous myxoma): a clinicopathologic study of 17 cases arising in the genital region. Int J Gynecol Pathol.

[10] Perret AG, Perrot JL, Dutoit M, Fouilloux B, Peoc'h M, Cambazard F (2005). Superficial angiomyxoma: report of four cases, including two subungueal tumors [Article in French]. Ann Pathol.

[11] Nakayama H, Hirol M, Kiyoku H, Naruse K, Enzan H (1997). Superficial angiomyxoma of the right inguinal region: report of a case. Jpn J Clin Oncol.

[12] Velanovich V (1996). Superficial angiomyxoma presenting as a groin hernia in a male toddler. Am Surg.

[13] Teixeira-De-Magalhães F, Pardal-De-Oliveira F (1995). Angiomyxoma of larynx. Report of one case of a myxoid fibrohistiocytic lesion. Pathologica.

[14] Izquierdo FM, Martin L, Burgos F, Lacruz C (1995). Fine-needle aspiration cytology of superficial angiomyxoma (myxoid perifollicular fibroma): report of a case. Diagn Cytopathol.

[15] Ustun C, Malazgirt Z, Kandemir B, Kocak I, Bolat I, Gumus S (1998). Anglomyofibroblastoma of the vulva: case report. Pathol Int.

[16] Bedlow AJ, Sampson SA, Holden CA (1997). Congenital superficial angiomyxoma. Clin Exp Dermatol.

[17] Chen YK, Lin LM, Lin CC, Yan YH (1998). Myxoid tumor of the oral cavity with features of superficial angiomyxoma: report of a case. J Oral Maxillofac Surg.

[18] Gardner AW (2007). Superficial angiomyxoma of the floor of the mouth—a case report. Br J Oral Maxillofac Surg.

[19] Yamamoto K, Kondo A, Iwashita K (2006). A case of superficial angiomyxoma. Tokai J Exp Clin Med.

[20] Rodríguez-Vázquez M, García-Arpa M, Delgado M, Cortina P, Vera E, Romero G (2005). Superficial angiomyxoma [Article in Spanish]. Actas Dermosifiliogr.

[21] Yuen HK, Cheuk W, Luk FO, Wat CS, Auyeung KC, Lam DS (2005). Solitary superficial angiomyxoma in the eyelid. Am J Ophthalmol.

[22] Okada Y, Mori H, Tsuji M, Yagi Y (2005). A case of vulvar superficial angiomyxoma with necrotizing angiitis-like lesions and expression of granulocyte-colony stimulating factor. Pathol Res Pract.

[23] Misago N, Mori T, Yoshioka M, Narisawa Y (2007). Digital superficial angiomyxoma. Clin Exp Dermatol.

[24] Wu SC, Lin SP (2006). Superficial angiomyxoma of neck: a case report [Article in Chinese]. Zhonghua Bing Li Xue Za Zhi.

[25] Kim HS, Kim GY, Lim SJ, Ki KD, Kim HC (2010). Giant superficial angiomyxoma of the vulva: a case report and review of the literature. J Cutan Pathol.

[26] Satter EK (2009). Solitary superficial angiomyxoma: an infrequent but distinct soft tissue tumor. J Cutan Pathol.

[27] Pérez Tato B, Sáez AC, Fernández PR (2008). Superficial angiomyxoma with trichofolliculoma. Ann Diagn Pathol.

[28] Meer S, Beavon I (2008). Intraoral superficial angiomyxoma. Oral Surg Oral Med Oral Pathol Oral Radiol Endod.

[29] Khadilkar UN, Khadilkar NP, Rao PS, Chakravorty S, Goel G (2007). Superficial angiomyxoma of the external ear not associated with Carney's complex: a case report. Kathmandu Univ Med J (KUMJ).

[30] Nakamura M, Tokura Y (2011). Superficial angiomyxoma on the scrotum of a child. Pediatr Dermatol.

[31] Rosado Rodríguez P, de Vicente JC, de Villalaín L, Blanco V (2012). Superficial angiomyxoma of the parotid region and review of the literature [Article in Spanish]. Acta Otorrinolaringol Esp.

[32] Zhu XH, Ou-Yang L, Li B, Ma NY (2010). Vulva superficial angiomyxoma: a case report. Zhejiang Da Xue Xue Bao Yi Xue Ban.

[33] Toth A, Nemeth T, Szucs A, Szollosi Z, Sziklai I (2010). Retropharyngeal superficial angiomyxoma. J Laryngol Otol.

[34] Qian P, Ma SR, Xu GT (2009). Superficial angiomyxoma: report of a case [Article in Chinese]. Zhonghua Bing Li Xue Za Zhi.

[35] Ali N, Child CS, Michaelides M, Olver JM (2011). Recurrence of a rare skin tumour: superficial angiomyxoma in the eyelid. Can J Ophthalmol.

[36] Falidas E, Rallis E, Vlachos C, Konstantoudakis S, Villias C (2011). Superficial subungual angiomyxoma: case report and review of the literature. J Cutan Med Surg.

[37] Basak S, Rogers S, Solomonsz AF (2011). Superficial angiomyxoma of the vulva: a case report of a rare cutaneous tumour. J Obstet Gynaecol.

[38] Nishio J, Iwasaki H, Aoki M, Nabeshima K, Naito M (2014). FDG PET/CT findings of superficial angiomyxoma. Clin Nucl Med.

[39] Sibley CD, Brown HA, Harrop AR, Haber RM (2013). Exophytic nodule on the scalp—quiz case. JAMA Dermatol.

[40] Zhu L, Zhao W, Shi Y, Lin B (2014). Superficial angiomyxoma of the vulva complicated with condyloma acuminatum and Staphylococcus hominis infection. Int J Dermatol.

[41] S V R, Raju MS, J D S, Taneja N, Chandra S, Mahajan S, Panwar E (2012). Intraoral superficial angiomyxoma of the upper alveolus: report of a unique case. Case Rep Med.

[42] Dınız G, Temır G, Ortaç R (2012). Angiomyxoma: always myxoid, sometimes aggressive [Article in Turkish]. Turk Patoloji Derg.

[43] Wang Z, Wei YB, Yin Z, Yan B, Li D, Zhou KQ, Yang JR (2015). Diagnosis and management of scrotal superficial angiomyxoma with the aid of a scrotoscope: case report and literature review. Clin Genitourin Cancer.

[44] Kahn SL, Juhl ME, Sidiropoulos M, Guitart J, Antonijevic S, Krunic AL (2016). Angiomyxoma of the nasal dorsum treated by Mohs surgery. Australas J Dermatol.

[45] Kura MM, Jindal SR (2014). Solitary superficial acral angiomyxoma: an infrequently reported soft tissue tumor. Indian J Dermatol.

[46] Green M, Logemann N, Sulit DJ (2014). Myxoid stroma and delicate vasculature of a superficial angiomyxoma give rise to the red planet sign. Dermatol Online J.

[47] Bhat AK, Acharya AM, Rosario P, Anuradha CK, Rao L (2014). Superficial angiomyxoma of the thumb mimicking a malignant bone tumor: case report. J Hand Surg Am.

[48] Zhang S, Xu ZQ (2013). Systemic lupus erythematosus complicated by superficial angiomyxoma in a child [Article in Chinese]. Zhongguo Dang Dai Er Ke Za Zhi.

[49] Lemtibbet S, Bourra H, Rimani M, Senouci K, Belgnaoui F, Hassam B (2013). Superficial plantar angiomyxoma [Article in French]. Ann Dermatol Venereol.

[50] Ozdemir M, Uzun I, Karahasanoglu A, Ceylan C (2016). A case of vulvar superficial angiomyxoma: a rare clinical entity. J Obstet Gynaecol.

[51] Victoria Martínez AM, Sánchez Carazo JL, Alegre de Miquel V (2016). Superficial angiomyxoma of the nipple: a case report of an infrequent cutaneous tumour. Dermatol Online J.

[52] Shukla S, Sehgal S, Prabhat P, Dewan K (2018). Congenital presentation of a solitary superficial angiomyxoma in the parotid region masquerading as parotid tumor. Turk Patoloji Derg.

[53] Lee SH, Cho YJ, Han M, Bae JW, Park JW, Oh SR, Kim S (2016). Superficial angiomyxoma of the vulva in a postmenopausal woman: a case report and review of literature. J Menopausal Med.

[54] Abarzúa-Araya A, Lallas A, Piana S, Longo C, Moscarella E, Argenziano G (2016). Superficial angiomyxoma of the skin. Dermatol Pract Concept.

[55] Aberdein G, Veitch D, Perrett C (2016). Mohs micrographic surgery for the treatment of superficial angiomyxoma. Dermatol Surg.

[56] Anehosur V, Adirajaiah S, Ghosh R (2016). Intraoral superficial angiomyxoma: a case report. J Maxillofac Oral Surg.

[57] Singhota S, Lam M, Gahir DS, Malins T (2017). Two rare cases of superficial angiomyxoma in the oral cavity. Br J Oral Maxillofac Surg.

[58] Wang YC, Li XM, Zhong GP, Xing Z, Wang ZP (2017). Superficial angiomyxoma of penis: a case report of a 6-year follow-up. Asian J Androl.

[59] Imen BS, Mounir M (2017). An unusual localisation of a superficial angiomyxoma. Pan Afr Med J.

[60] Bajpai M, Pardhe N, Vijay P (2017). Superficial angiomyxoma of palate. J Coll Physicians Surg Pak.

[61] Hamzelou S, Ghanadan A, Daneshpazhooh M, Kiani A, Mahmoudi H (2017). Superficial plantar angiomyxoma in a young man. Australas J Dermatol.

[62] O'Flynn O'Brien KL, Cortes-Santiago N, Patil NM, Bercaw-Pratt JL (2020). A rare case of vulvar superficial angiomyxoma in a pediatric patient. J Pediatr Adolesc Gynecol.

[63] Iwashita W, Kurabayashi A, Tanaka C, Naganuma S, Kawamura T, Aki F, Furihata M (2020). Superficial angiomyxoma of the nipple in a japanese woman: a case report and review of literature. Int J Surg Pathol.

[64] Chen L, Chen L, Wang JL, Hu C, Liu ZX, Yuan XC, He JX (2019). Sonographic findings in superficial angiomyxoma of the vulva in a perimenopausal female. J Med Ultrasound.

[65] Hafeez F, Krakowski AC, Lian CG, Nazarian RM, Maleszewski JJ (2022). Sporadic superficial angiomyxomas demonstrate loss of PRKAR1A expression. Histopathology.

[66] Hwang YJ, Lee HW, Lee IS, Jung SG, Lee HK (2021). Superficial angiomyxoma of the posterior neck. Arch Craniofac Surg.

[67] Amores-Martín E, de Los Ángeles Sola Casas M, Fernández-Figueras MT (2021). Superficial angiomyxoma: dermoscopic findings [Article in English, Spanish]. Actas Dermosifiliogr (Engl Ed).

[68] Chen X, Ma S, Li Y, Xu B, Liu Z (2022). Superficial angiomyxoma in nasal vestibule: a case report [Article in Chinese]. Lin Chuang Er Bi Yan Hou Tou Jing Wai Ke Za Zhi.

[69] Hosapatna Basavarajappa D, Gainder S, Srinivasan R (2022). Giant superficial angiomyxoma of the vulva - a bizarre presentation. J Obstet Gynaecol Can.

[70] Morimoto M, Takano M, Sato T, Kitamura T, Makino S (2022). Rapidly growing superficial angiomyxoma in mandibular gingiva: a case report and literature review. Head Neck Pathol.

[71] Fotiadou S, Garefis K, Chatziavramidis A, Konstantinidis I, Massa E, Markou K, Konstantinidis I (2021). Large superficial angiomyxoma of the tongue causing dysphagia. Ear Nose Throat J.

[72] Dubin I, Mortazavi S, Yu T, Riahi IR, Baker JL (2021). Superficial angiomyxoma of the breast in a 16-year-old girl without carney's complex: a case report. Breast J.

[73] Xu Y, Duan Y, Zhou H, Chen H, Yan W (2023). Ultrasonic features of superficial angiomyxoma in the scrotum: a case image. J Clin Ultrasound.

[74] Chen G, Liu W, Cao Y, Guo X, Xie Y (2023). Imaging findings of superficial angiomyxoma in the eye socket. Clin Case Rep.

[75] Meng T, Yin H, Chen L, Zhao Q (2023). Superficial angiomyxoma of the breast in a 25-year-old woman without Carney complex. Asian J Surg.

[76] Yan S, Zou Y, Liao X (2022). Giant superficial angiomyxoma of the male perineum: a case report. Front Surg.

[77] Chijiiwa Y, Nagano T, Nishio J (2023). Superficial angiomyxoma of the wrist: case report and literature review. In Vivo.

[78] Hirai H, Kayamori K, Noji R, Kuroshima T, Ikeda T, Harada H (2023). A rare case of solitary intraoral superficial angiomyxoma arising in the soft palate. J Oral Sci.

[79] Navea OV, Navea MB, De la Fuente R (2023). Superficial angiomyxoma in an uncommon area: a case report. Cureus.

[80] Tonape T, Sv S (2023). Superficial angiomyxoma of axilla: a case report. Cureus.

[81] Park HK, Yun SJ, Lee SC, Lee JB (2023). A case of superficial angiomyxoma localized on the nipple in a 12-year-old Korean female. Ann Dermatol.

[82] Singh A, Rawat S, Kumar G, Singh US, Sagar M (2023). Solitary superficial angiomyxoma of the ear pinna: a diagnostic dilemma with a review of literature. Arch Clin Cases.

[83] Navitski A, Adams L, Brzezinska BN (2023). A tale of two vulvar angiomyxomas: two cases and review of literature. Gynecol Oncol Rep.

[84] Mehrotra K, Bhandari M, Khullar G, Sharma S (2021). Large superficial angiomyxoma of the vulva-report of two cases with varied clinical presentation. Indian Dermatol Online J.

[85] Yun YI, Lee KS, Khwarg SI, Kim N (2020). Rare case of isolated superficial angiomyxoma of the eyelid. Korean J Ophthalmol.

[86] Bembem K, Jaiswal A, Singh M, Verma N, Jain S, Bhat A (2017). Cyto-histo correlation of a very rare tumor: superficial angiomyxoma. J Cytol.

[87] Lee CC, Chen YL, Liau JY, Chen CA, Cheng WF (2014). Superficial angiomyxoma on the vulva of an adolescent. Taiwan J Obstet Gynecol.

[88] Quatresooz P, Hermanns JF, Legrain A, Piérard GE (2004). Clinical case of the month. Superficial angiomyxoma [Article in French]. Rev Med Liege.

[89] Moher D, Liberati A, Tetzlaff J, Altman DG (2009). Preferred reporting items for systematic reviews and meta-analyses: the PRISMA statement. PLoS Med.

[90] Calonje JE, Brenn T, Lazar A, Billings SD (2018). McKee’s Pathology of the Skin, 5th Edition. https://shop.elsevier.com/books/mckees-pathology-of-the-skin/calonje/978-0-7020-6983-3.

